# Supplementary material for: Constructing robust heterostructured interface for anode-free zinc batteries with ultrahigh capacities
Source: Nat Commun. 2023 Jan 5;14:76. doi: 10.1038/s41467-022-35630-6 (PMC9816316; doi:10.1038/s41467-022-35630-6)
Supplement: Supplementary file 1 — Supplementary Information [file 41467_2022_35630_MOESM1_ESM.docx]

Supplementary Information

**Constructing robust heterostructured interface for anode-free zinc batteries with ultrahigh capacities**

Xinhua Zheng^1,#^, Zaichun Liu^1,#^, Jifei Sun^1^, Ruihao Luo^1^, Kui Xu^1^, Mingyu Si^2^, Ju Kang^2^, Yuan Yuan^1^, Shuang Liu^1^, Touqeer Ahmad^1^, Taoli Jiang^1^, Na Chen^1^, Mingming Wang^1^, Yan Xu^1^, Mingyan Chuai^1^, Zhengxin Zhu^1^, Qia Peng^1^, Yahan Meng^1^, Kai Zhang^1^, Weiping Wang^1^, Wei Chen^1,^*

^1^Department of Applied Chemistry, School of Chemistry and Materials Science, Hefei National Research Center for Physical Sciences at the Microscale, University of Science and Technology of China, Hefei, Anhui 230026, China

^2^School of Mechanical Engineering, Beijing Institute of Petrochemical Technology, Beijing 102617, China

^#^These authors contributed equally to this work.

*Corresponding author. E-mail address*:* [weichen1@ustc.edu.cn](mailto:weichen1@ustc.edu.cn)

**Supplementary Figures**

**
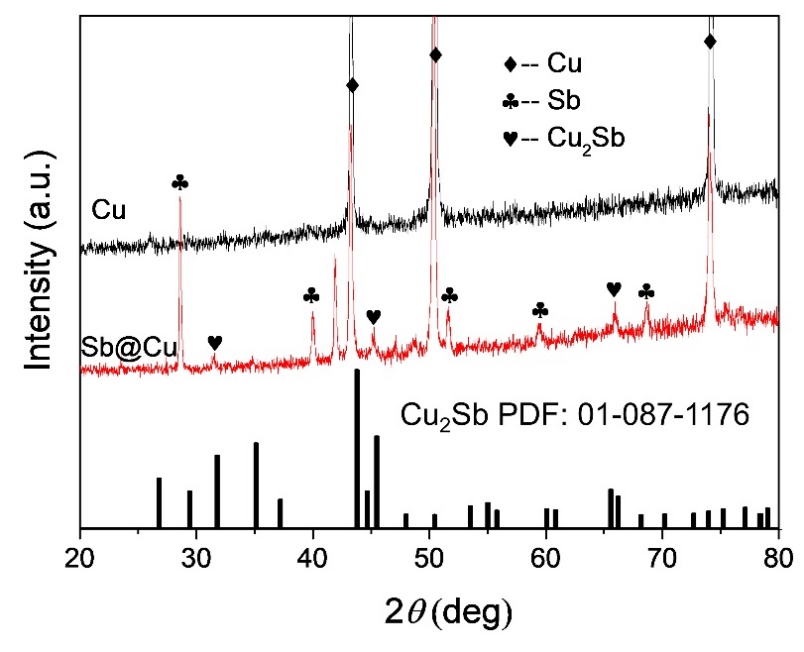
**

**Supplementary Fig. 1 |** XRD patterns of the substrates, including Cu foil and Sb plated Cu foil. The Sb plating on Cu with a current density of 3 mA cm^-2^ for 10 min.


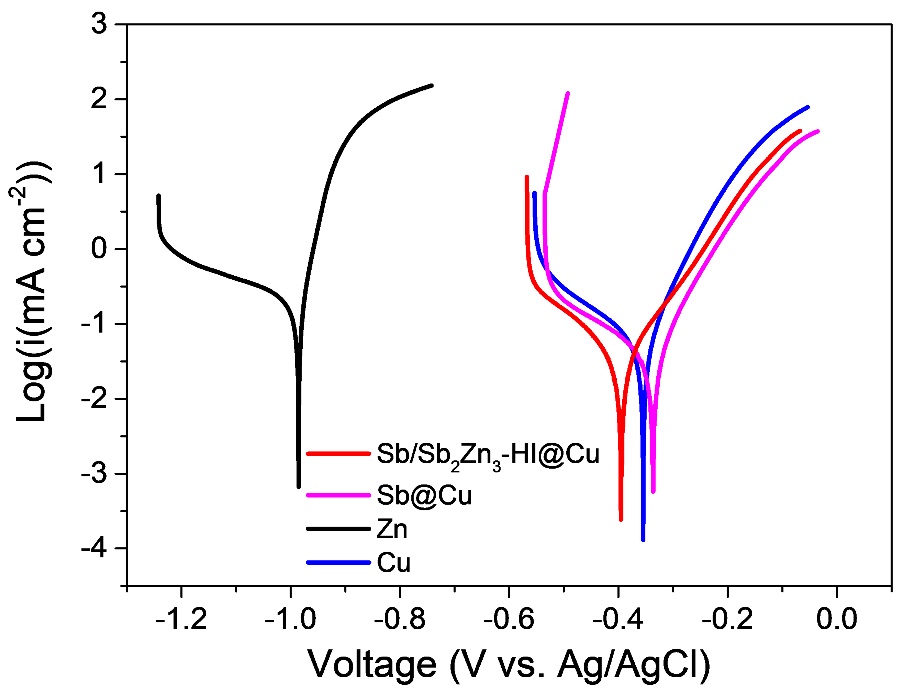


**Supplementary Fig. 2 |** Tafel plots of the Zn, Cu, Sb@Cu and Sb/Sb_2_Zn_3_-HI@Cu (Sb@Cu plated by 0.2 mAh cm^-2^ of Zn) substrates in 2 M KBr with a scan rate of 5 mV/s. The corrosion current density of the substrates of Zn, Cu, Sb@Cu and Sb/Sb_2_Zn_3_-HI@Cu are 0.21 mA cm^-2^, 0.072 mA cm^-2^, 0.049 mA cm^-2^ and 0.025 mA cm^-2^, respectively. The electrochemical measurements were carried out at room temperature (25 ℃).


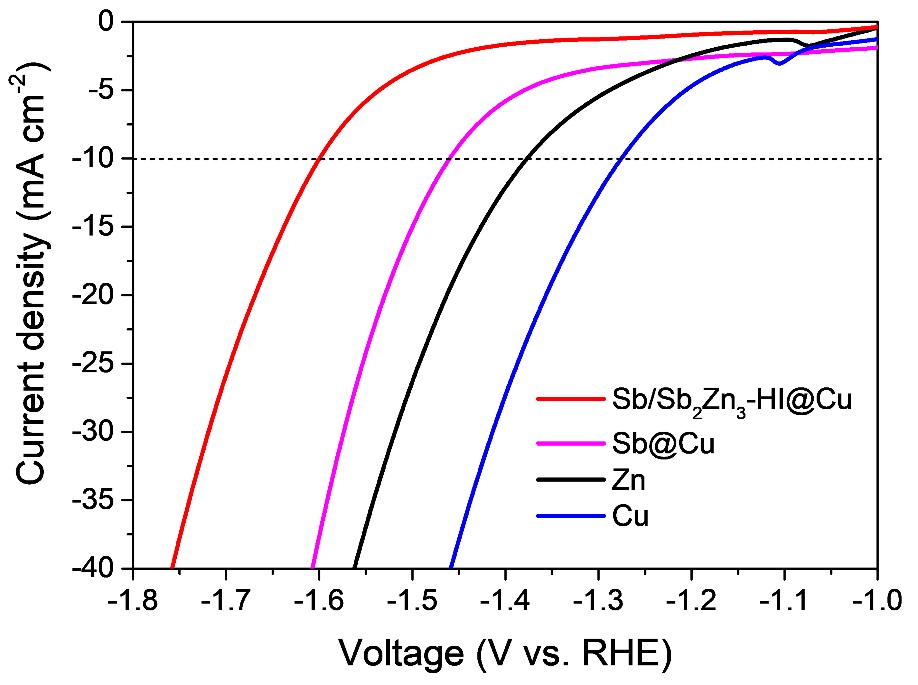


**Supplementary Fig. 3 |** HER polarization curves of the substrates including Zn, Cu, Sb@Cu and Sb/Sb_2_Zn_3_-HI@Cu (Sb@Cu plated by 0.2 mAh cm^-2^ of Zn) with a scan rate of 5 mV/s. The electrochemical measurements were carried out at room temperature (25 ℃).

**
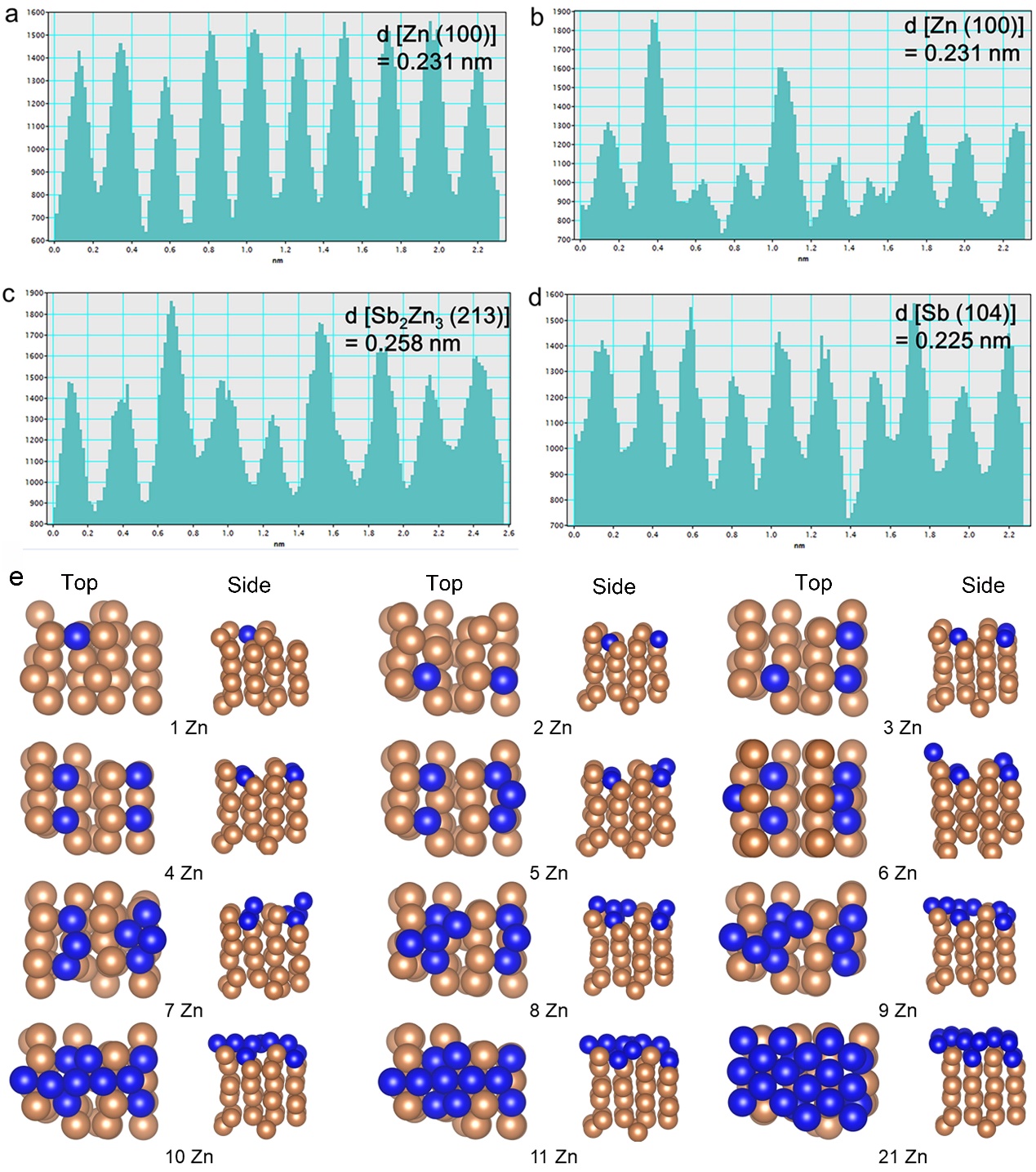
**

**Supplementary Fig. 4 |** Lattice spacing analysis of the Zn deposited Sb@Cu anode. **a** Zn (100) in region 1, **b** Zn (100) in region 2, **c** Sb_2_Zn_3_ (213), **d** Sb (104). The sample was treated by scraping off the surface layer of Zn plated with 0.2 mAh cm^-2^ on Sb@Cu, then put in ethanol for 30 min of ultrasonication, and finally dropped onto the copper TEM grid. **e** Optimized structures with the different numbers (1~11, 21) of adsorbed Zn on the Sb (104).


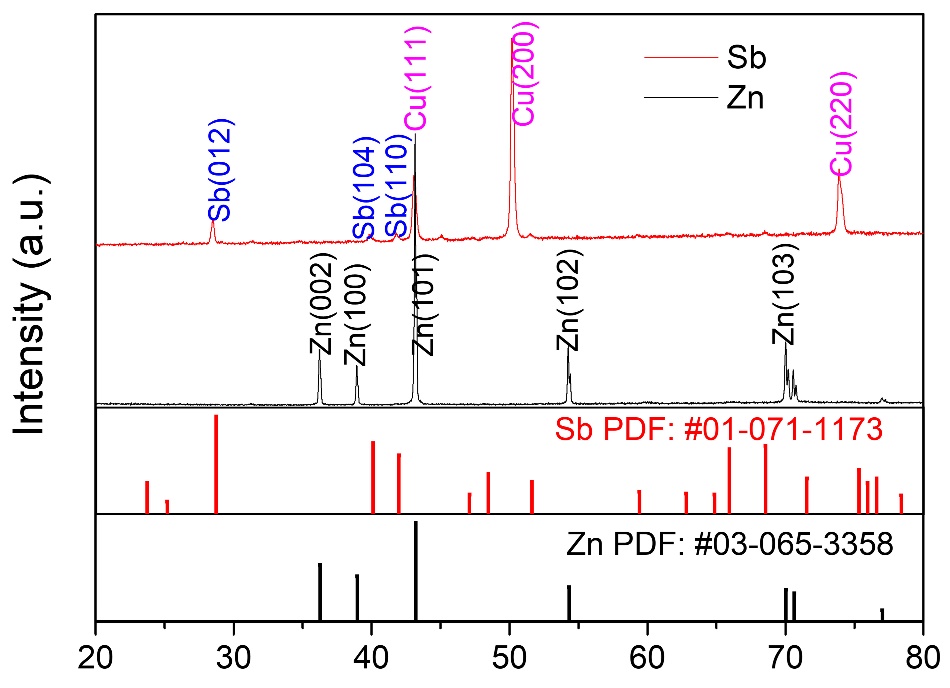


**Supplementary Fig. 5 |** XRD patterns of the Zn (Zn plated on Zn foil with 10 mAh cm^-2^) and Sb@Cu (Sb plated on Cu for 10 min at 3 mA cm^-2^) substrates.


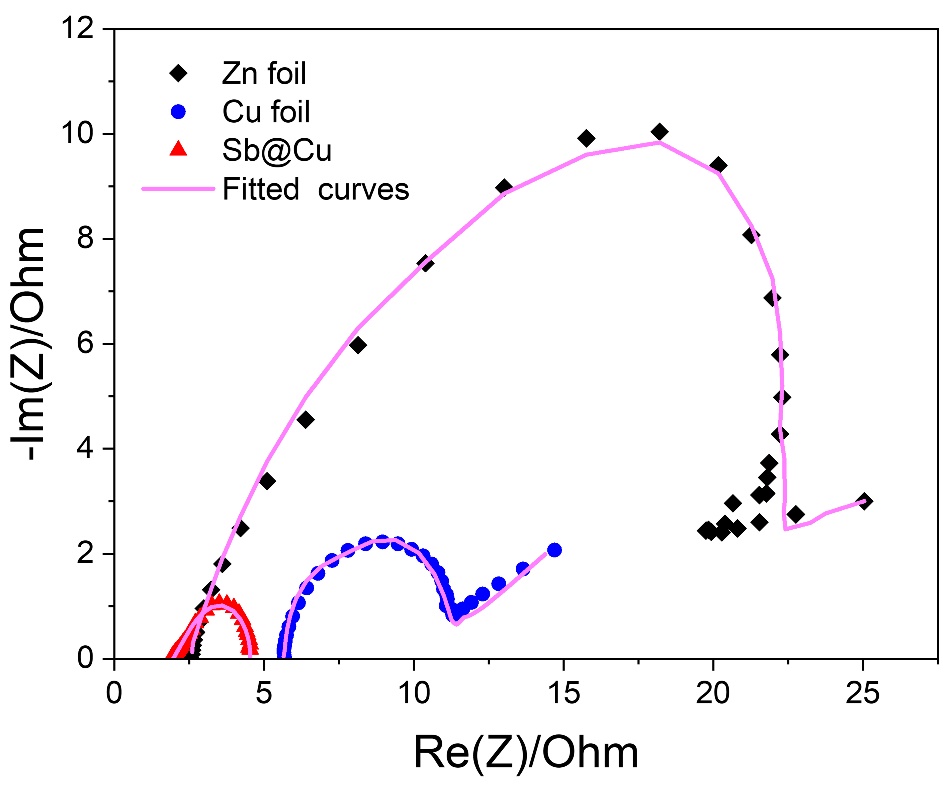


**Supplementary Fig. 6 |** Electrochemical impedance spectroscopy and corresponding fitted curves of the Zn, Cu and Sb@Cu substrates in 2 M ZnBr_2_. The electrochemical measurements were carried out at room temperature (25 ℃).


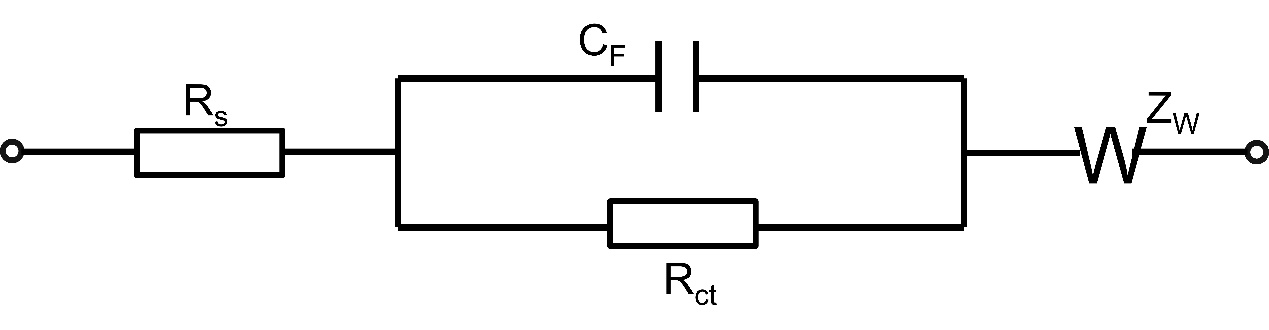


**Supplementary Fig. 7 |** An equivalent circuit is used to simulate the resistances of different substrates, where R_s_ is the ohmic resistance of solution and electrodes, R_ct_ is the charge transfer resistance, C_F_ is the double-layer capacitance, and Z_W_ is the Warburg impedance.


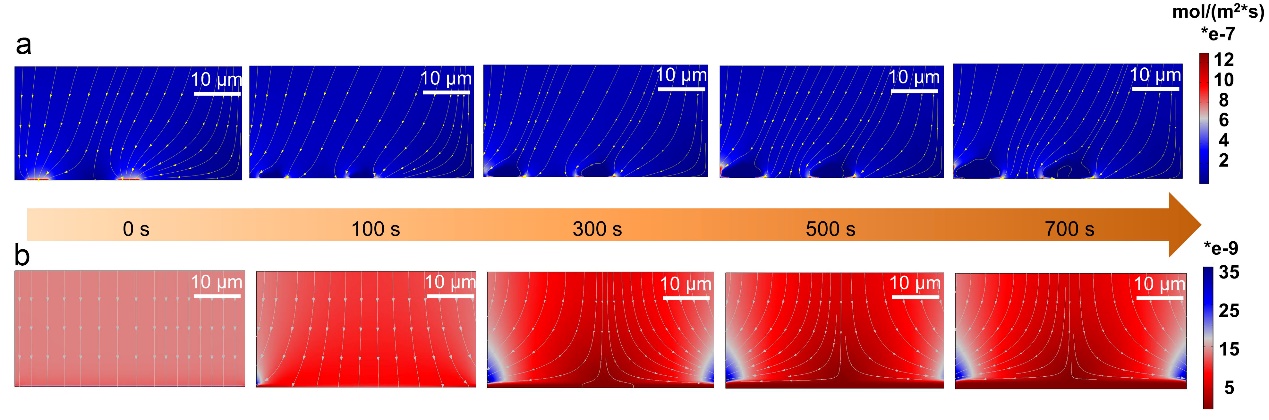


**Supplementary Fig. 8 | Simulated Zn^2+^ flux distribution at different Zn electrodeposition durations.** **a** Zn electrodeposition on Zn foil. **b** Zn electrodeposition on Sb/Sb_2_Zn_3_-HI@Cu substrate. The simulation processes were carried out at room temperature (25 ℃)


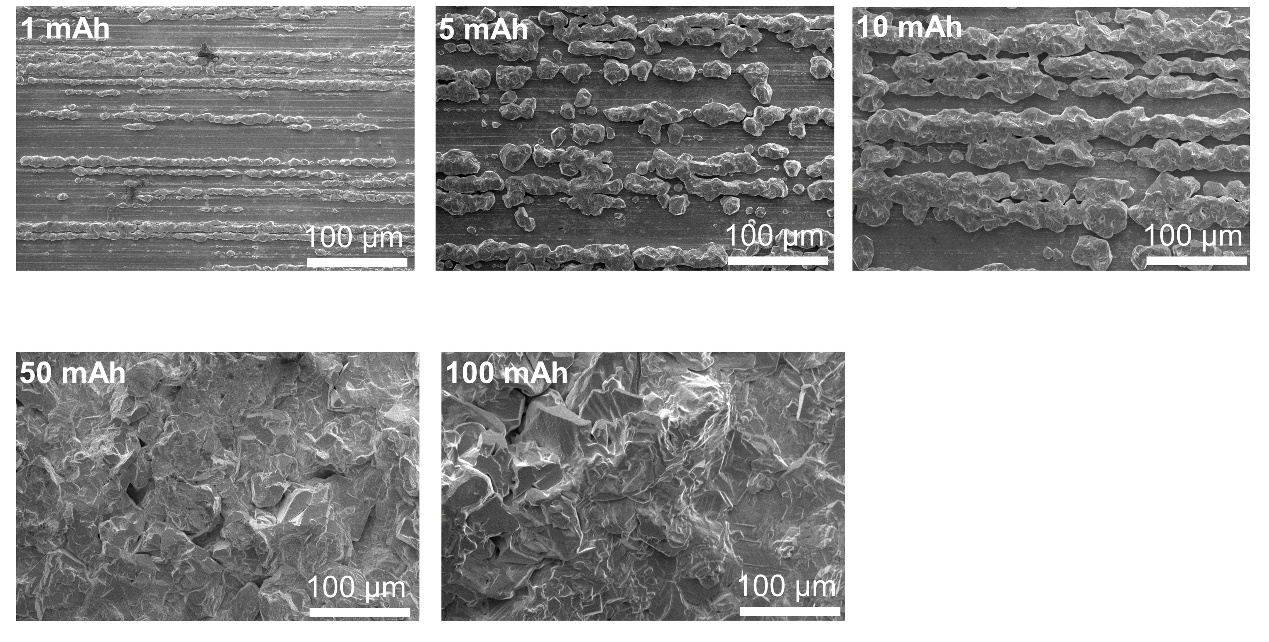


**Supplementary Fig. 9 |** SEM images of the Zn electroplating on Cu at the capacities from 1 to 100 mAh cm^-2^.


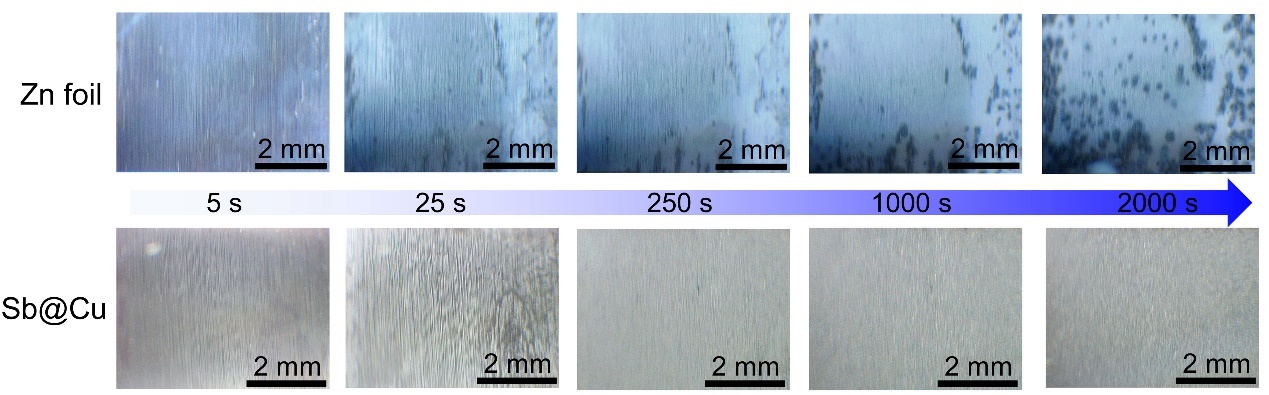


**Supplementary Fig. 10 |** In-situ observation of morphologies of the Zn electrodeposition on Zn foil and Sb@Cu substrates in 2 M ZnBr_2_ at a current density of 20 mA cm^-2^. The electroplating measurements were carried out at room temperature (25 ℃).

**
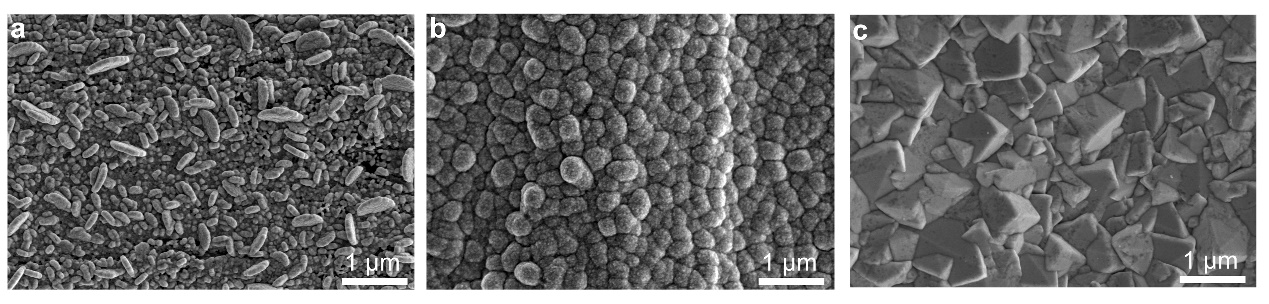
**

**Supplementary Fig. 11 |** SEM images of Sb plating on Cu foils under a constant current of 3 mA cm^-2^ with different times. **a** 3 min. **b** 10 min. **c** 20 min. The Cu surface plated by Sb for 10 min showed a uniform and dense surface, and the nano-spherical Sb metal distributed on the surface provided a larger specific surface area, which facilitated the deposition of Zn.

**
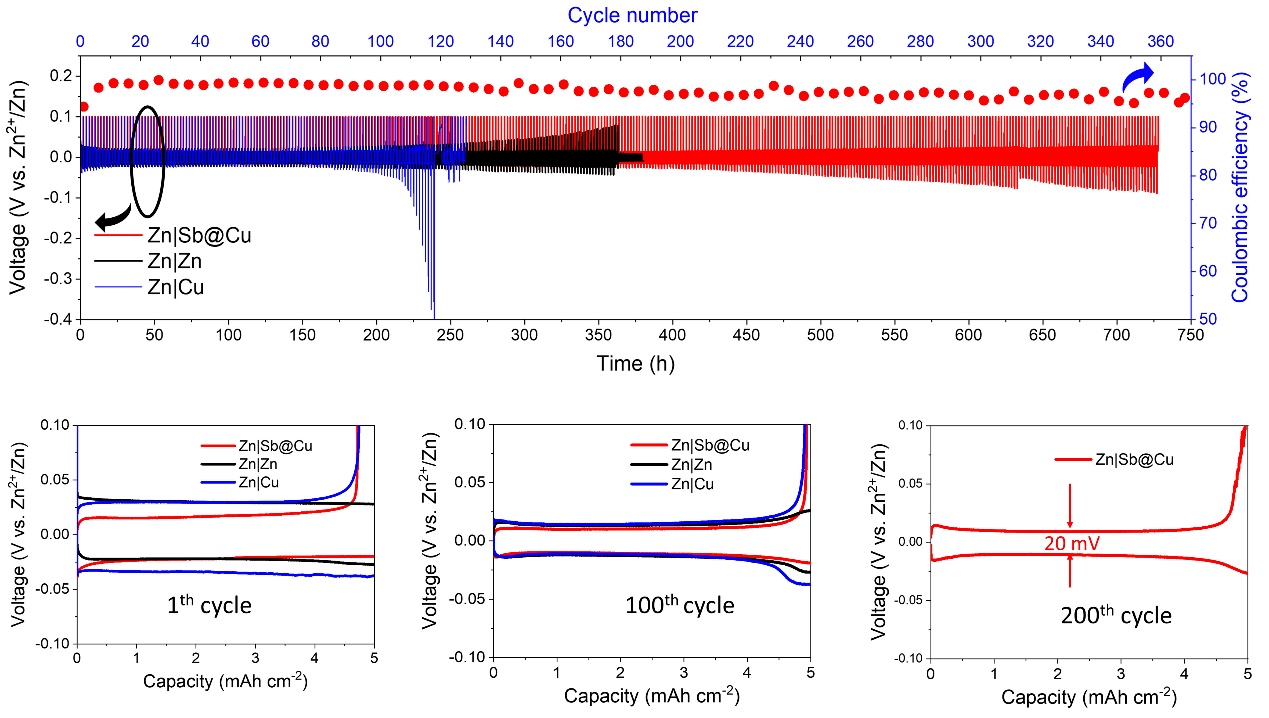
**

**Supplementary Fig. 12 |** Electrochemical performance of symmetric Zn|Zn and asymmetric Zn|Cu and Zn|Sb@Cu half-cells in coin configurations. The cells were charged and discharged with an areal capacity of 5 mAh cm^-2^ and a current density of 5 mA cm^-2^, while the discharge cut off voltage were 0.1 V vs. Zn^2+^/Zn. The electrochemical measurements were carried out at room temperature (25 ℃).

**
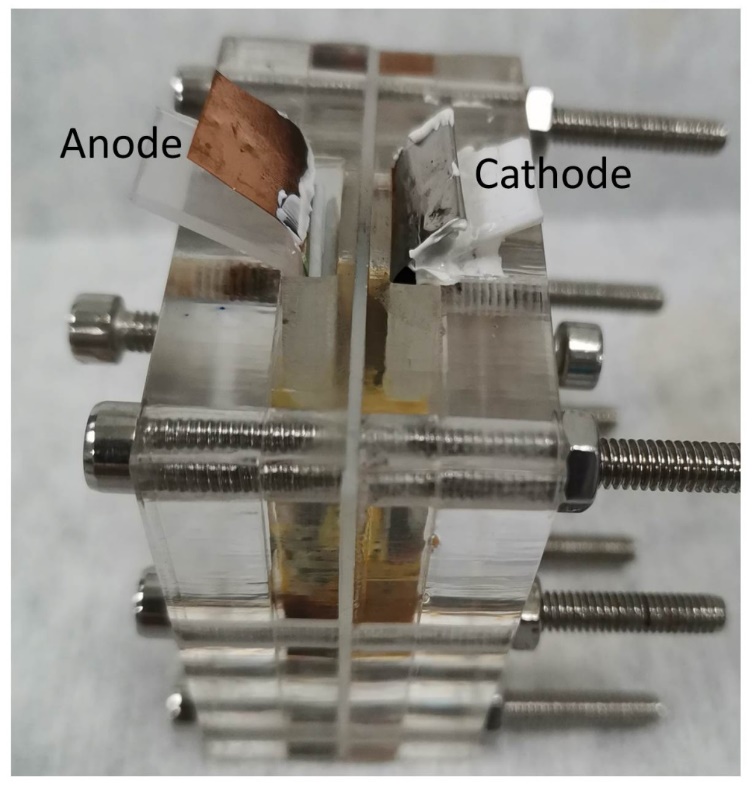
**

**Supplementary Fig. 13 |** A digital photograph of the homemade plexiglass device used for Zn-Br_2_ battery tests.


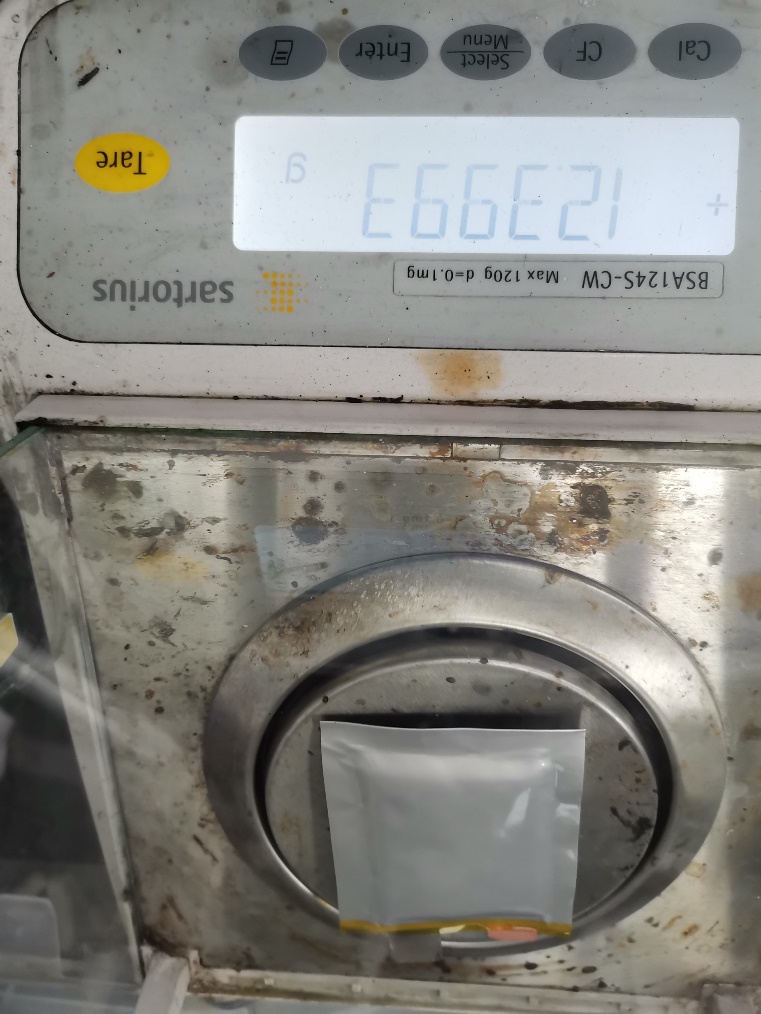


**Supplementary Fig. 14 |** A digital photo of the weight of the pouch cell measured by an electric microbalance.


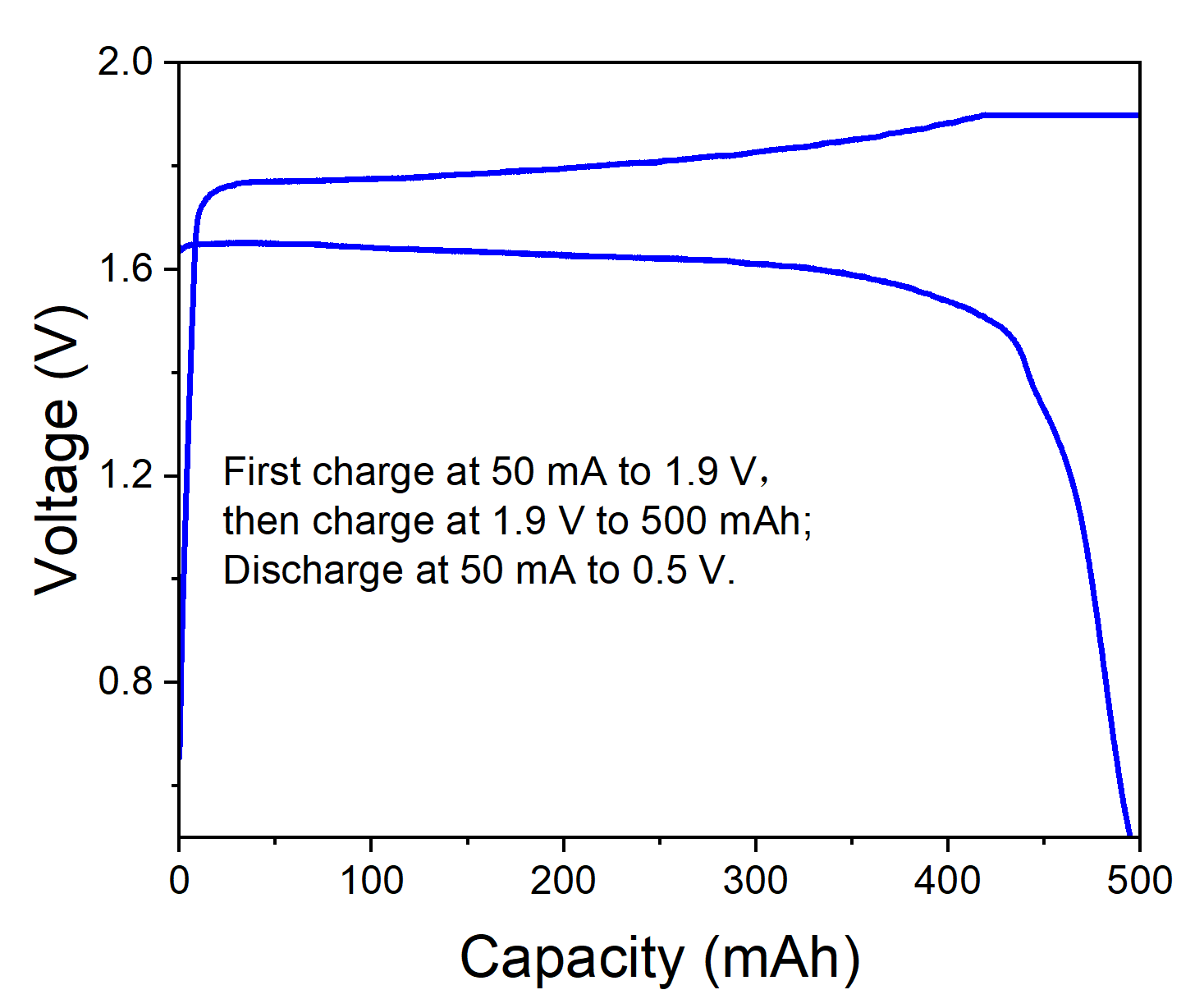


**Supplementary Fig. 15 |** Charge and discharge curves of the pouch cell for practical energy density calculation.

We have further optimized the scaled-up Zn-Br_2_ battery under real conditions for practical energy density calculation. The 500 mAh scaled-up battery was built in a pouch cell, where the electrode sizes were about 4.5 cm * 3.5 cm with an areal capacity of 31.7 mAh cm^-2^. Specifically, the optimized scaled-up battery consists of cathode, anode, electrolyte, separator, current collectors and cell packaging. The total weight of the battery is about 12.4 g (Supplementary Fig. 14). The N/P ratio of the battery is about 1.25:1, and the electrolyte is used to wet the separator and electrodes. The charge and discharge curves of the pouch cell with the obtained discharge energy of 766.9 mWh are shown in Supplementary Fig. 15. Therefore, the battery shows a practical energy density of about 62 Wh kg^-1^ based on the total mass of the whole pouch cell. We believe that the energy density of our battery can be largely increased upon further optimization in future study.

**
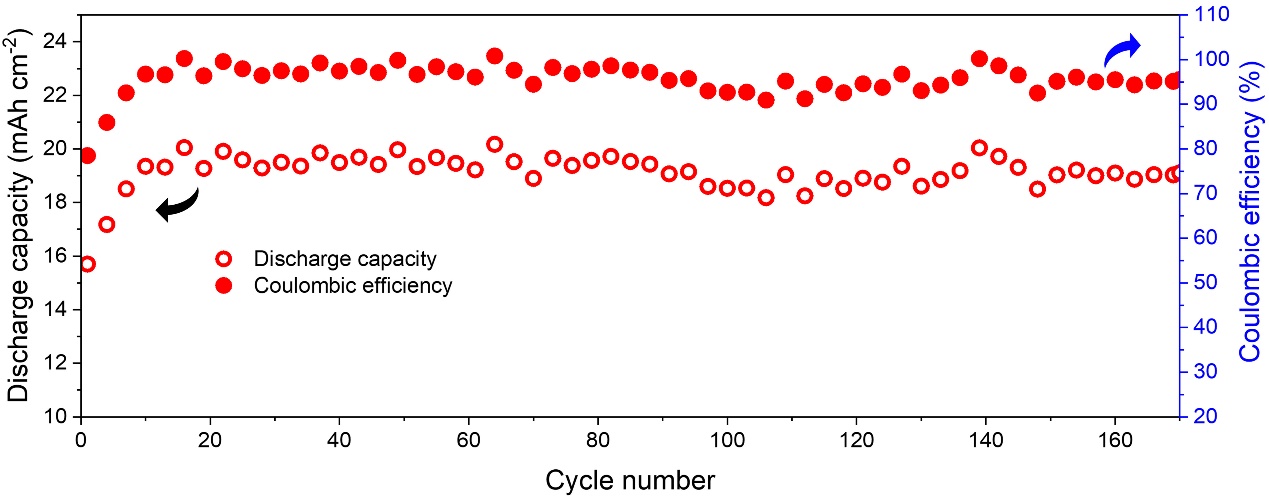
**

**Supplementary Fig. 16 |** Electrochemical performance of the Zn-Br_2_ batteries with an areal capacity of 20 mAh cm^-2^, a current density of 20 mA cm^-2^, and a discharge cut off voltage of 0.5 V. The electrochemical measurement was carried out at room temperature (25 ℃).

**
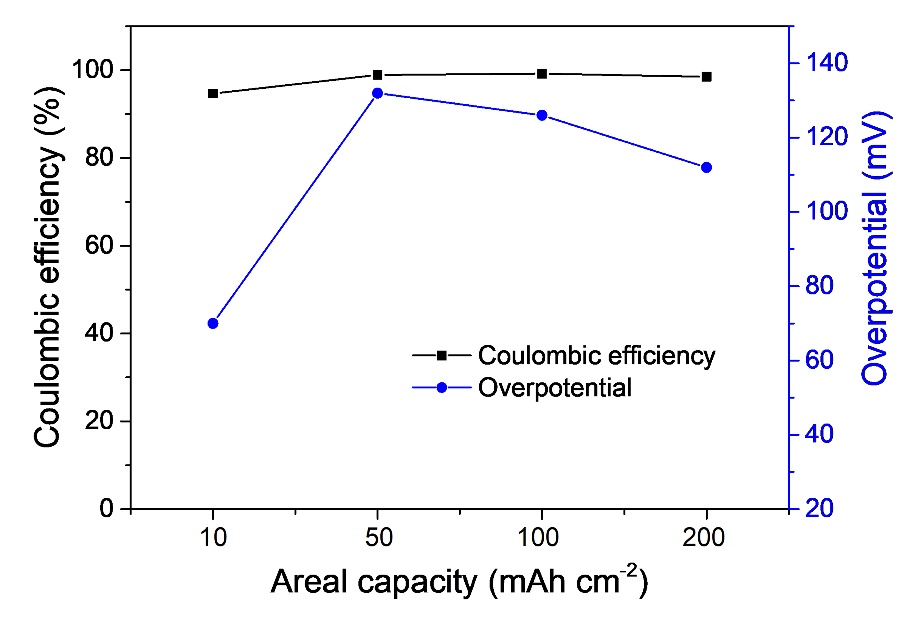
**

**Supplementary Fig. 17 |** Overpotential and CE values of the Zn|Sb@Cu half-cells at different capacities.

**
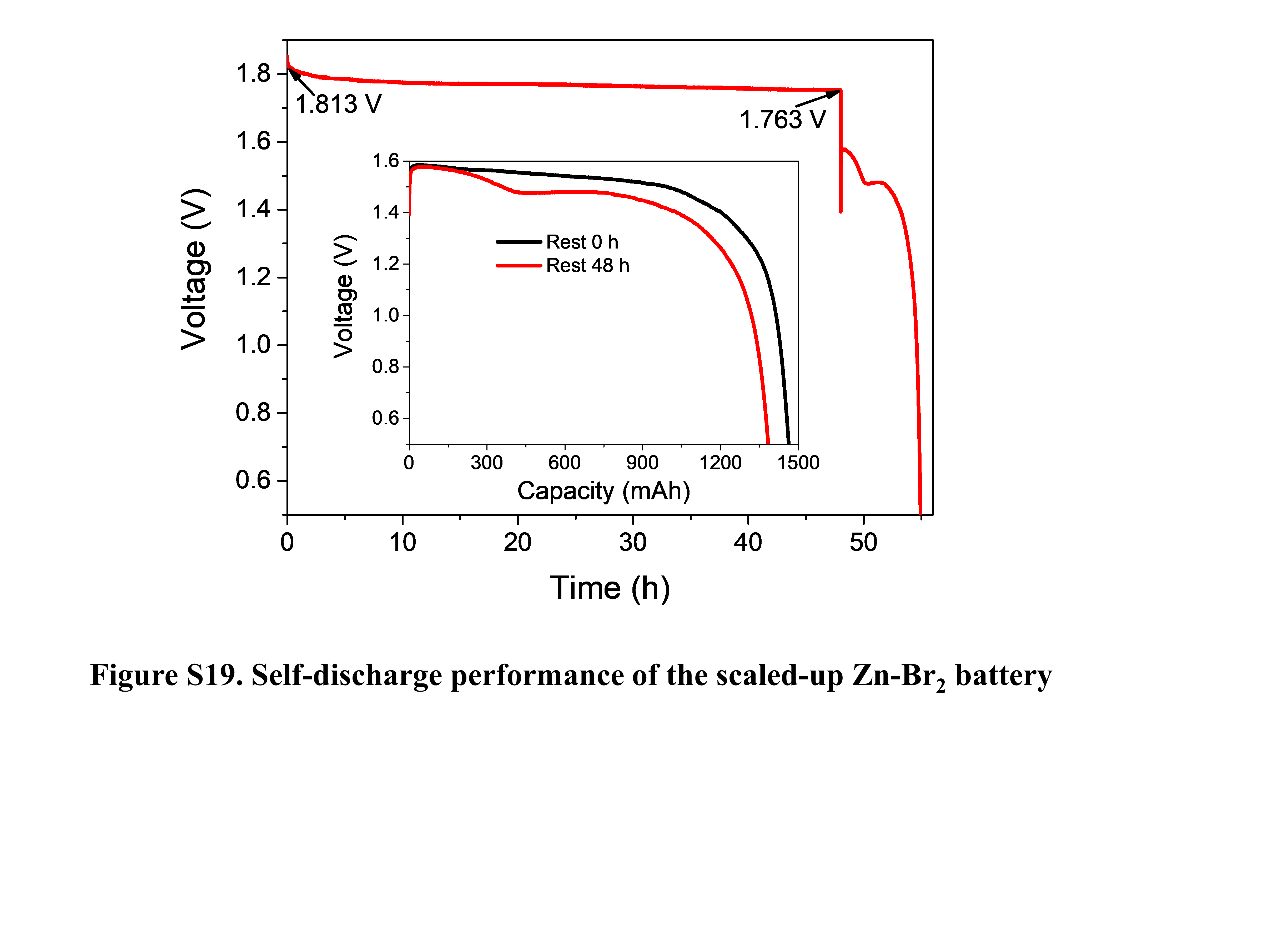
**

**Supplementary Fig. 18 |** Self-discharge performance of the scaled-up Zn-Br_2_ battery. The battery was charged firstly at 300 mA to 2 V and then at 2 V to 1500 mAh, and after a rest period it was discharged at 200 mA to 0.5 V. The electrochemical measurements were carried out at room temperature (25 ℃).

**
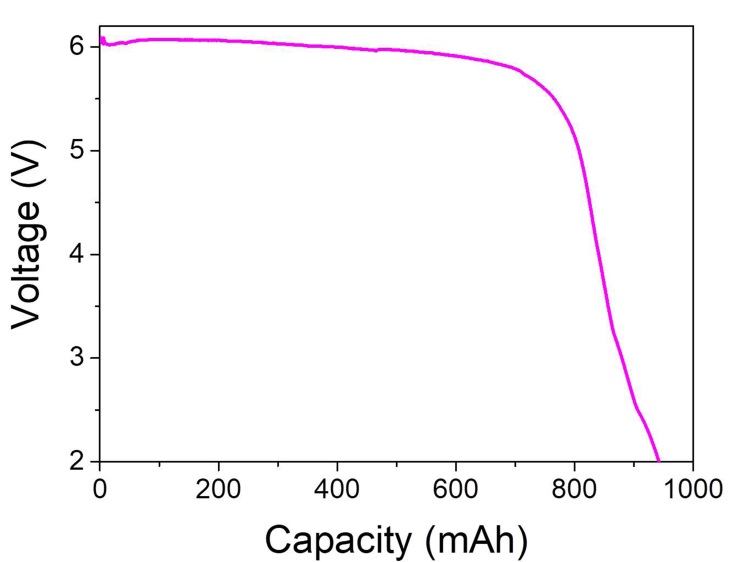
**

**Supplementary Fig. 19 |** Discharge curves of the four series-connected Zn-Br_2_ battery module, where the battery was charged using photovoltaic cell panel and have been illuminated with 10 W LEDs for 10 minutes before being discharged at 200 mA to 2 V. The electrochemical measurement was carried out at room temperature (25 ℃).

**Supplementary Table 1 |** Summary of electrochemical performance of the anode-free Zn batteries in terms of different parameters.

| **Anode** | **Electrolyte** | **Current density (mA cm^-2^)** | **Maximum areal capacity**  **(mAh cm^-2^)** | **Retention/Cycles or time**  **(Areal capacity, mAh cm^-2^, Discharge current density, mA cm^-2^)** | **Ref.** |
| --- | --- | --- | --- | --- | --- |
| **Sb@Cu** | **2 M ZnBr_2_** | **200** | **200** | **98%/550 h**  **(10 mAh cm^-2^, 20 mA cm^-2^)** | **This work** |
| C@Cu | 3 M Zn(CF_3_SO_3_)_2_ | 2 | 3 | 100%/300  (0.5 mAh cm^-2^, 1 mA cm^-2^) | [^1^](#_ENREF_1) |
| MOF-ZIF-8 | 2 M ZnSO_4_ | 30 | 10 | 72%/20000  (4 A g^-1^) | [^2^](#_ENREF_2) |
| Cu nanowire | 2 M ZnSO_4_ | 10 | 5 | 100%/130  (5 mAh cm^-2^, 10 mA cm^-2^) | [^3^](#_ENREF_3) |
| Ag@Cu | 3 m Zn(TFSI)_2_/EMC | 0.5 | 0.5 | 99.86%/200  (0.5 mAh cm^-2^, 0.5 mA cm^-2^) | [^4^](#_ENREF_4) |
| 3D Cu | 2 M ZnSO_4_ | 1 | 2 | 100/350 h  (0.5 mAh cm^-2^, 0.5 mA cm^-2^) | [^5^](#_ENREF_5) |
| CNT@CC | 2 M ZnSO_4_ | 5 | 2 | 100/200 h  (2 mAh cm^-2^, 2 mA cm^-2^) | [^6^](#_ENREF_6) |
| Sn@CF | 2 M ZnBr_2_ | 40 | 40 | 99.4/290  (20 mAh cm^-2^, 40 mA cm^-2^) | [^7^](#_ENREF_7) |
| 3D Ti-TiO_2_ | 2 M ZnSO_4_ | 10 | 5 | 93.69/200  (5 mAh cm^-2^, 10 mA cm^-2^) | [^8^](#_ENREF_8) |
| MXene@Sb | 2 M ZnSO_4_ | 10 | 2 | ~90%/300  (2 mAh cm^-2^, 10 mA cm^-2^) | [^9^](#_ENREF_9) |
| F-rich interfacial@Zn foil | 2 M ZnSO_4_ + 0.08 M ZnF_2_ | 40 | 3 | 99.87/600 h  (1 mAh cm^-2^, 1 mA cm^-2^) | [^10^](#_ENREF_10) |
| Ag mesh | 2 M ZnSO_4_ | 10 | 5 | 99.5%/2000  (1 mAh cm^-2^, 5 mA cm^-2^) | [^11^](#_ENREF_11) |

**Supplementary Table 2 |** The fitting results of EIS plots of all the samples in 2 M ZnBr_2_.

| Samples | R_s_ | R_ct_ | C_F_ |
| --- | --- | --- | --- |
| Sb@Cu | 1.91 | 2.62 | 0.017 |
| Cu | 5.82 | 4.99 | 5.55e-5 |
| Zn | 2.56 | 19.78 | 3.06e-5 |

**Supplementary Table 3** | The physical parameters for COMSOL simulation.

| Parameters | Values | Descriptions |
| --- | --- | --- |
| cbZn | 2000 mol/m^3^ | Bulk concentration of Zn |
| rhoZn | 7140 kg/m³ | Density of Zn |
| DZn | 7E-10 m²/s | Diffusion coefficient of Zn |
| MZn | 0.06538 kg/mol | Molar mass of Zn |
| T | 293.15 K | Temperature |

**Supplementary Reference**

1. Zhu Y., Cui Y. & Alshareef H. N. An anode-free Zn–MnO_2_ battery. *Nano Lett.* **21**, 1446-1453 (2021).

2. Wang Z.*, et al.* A metal-organic framework host for highly reversible dendrite-free zinc metal anodes. *Joule* **3**, 1289-1300 (2021).

3. Xie S.*, et al.* Stable zinc anodes enabled by zincophilic Cu nanowire networks. *Nano-Micro Lett.* **14**, 39 (2021).

4. Wang G.*, et al.* An anode-free Zn-graphite battery. *Adv. Mater.* **34**, 2201957 (2022).

5. Kang Z.*, et al.* 3D porous copper skeleton supported zinc anode toward high capacity and long cycle life zinc ion batteries. *ACS Sustain. Chem. Eng.* **7**, 3364-3371 (2019).

6. Zeng Y.*, et al.* Dendrite-free zinc deposition induced by multifunctional CNT frameworks for stable flexible Zn-ion batteries. *Adv. Mater.* **31**, 1903675 (2019).

7. Yin Y.*, et al.* Dendrite-free zinc deposition induced by tin-modified multifunctional 3D host for stable zinc-based flow battery. *Adv. Mater.* **32**, 1906803 (2020).

8. An Y., Tian Y., Xiong S., Feng J. & Qian Y. Scalable and controllable synthesis of interface-engineered nanoporous host for dendrite-free and high rate zinc metal batteries. *ACS Nano* **15**, 11828-11842 (2021).

9. Tian Y., An Y., Liu C., Xiong S., Feng J. & Qian Y. Reversible zinc-based anodes enabled by zincophilic antimony engineered MXene for stable and dendrite-free aqueous zinc batteries. *Energy Storage Mater.* **41**, 343-353 (2021).

10. An Y.*, et al.* Stable aqueous anode-free zinc batteries enabled by interfacial engineering. *Adv. Funct. Mater.* **31**, 2101886 (2021).

11. Xue R.*, et al.* Highly reversible zinc metal anodes enabled by a three-dimensional silver host for aqueous batteries. *J. Mater. Chem. A* **10**, 10043-10050 (2022).
